# Fine particulate matter (PM_2.5_) inhalation-induced alterations in the plasma lipidome as promoters of vascular inflammation and insulin resistance

**DOI:** 10.1152/ajpheart.00881.2020

**Published:** 2021-03-05

**Authors:** Bradford G. Hill, Benjamin Rood, Amanda Ribble, Petra Haberzettl

**Affiliations:** ^1^Diabetes and Obesity Center, Christina Lee Brown Envirome Institute, Division of Environmental Medicine, Department of Medicine, University of Louisville, Louisville, Kentucky;; ^2^Department of Biochemistry and Molecular Genetics, University of Louisville, Louisville, Kentucky

**Keywords:** air pollution, cardiovascular disease, free fatty acids, plasma metabolome, pulmonary oxidative stress

## Abstract

Fine particulate matter (PM_2.5_) air pollution exposure increases the risk of developing cardiovascular disease (CVD). Although the precise mechanisms by which air pollution exposure increases CVD risk remain uncertain, research indicates that PM_2.5_-induced endothelial dysfunction contributes to CVD risk. Previous studies demonstrate that concentrated ambient PM_2.5_ (CAP) exposure induces vascular inflammation and impairs insulin and vascular endothelial growth factor (VEGF) signaling dependent on pulmonary oxidative stress. To assess whether CAP exposure induces these vascular effects via plasmatic factors, we incubated aortas from naïve mice with plasma isolated from mice exposed to HEPA-filtered air or CAP (9 days) and examined vascular inflammation and insulin and VEGF signaling. We found that treatment of naïve aortas with plasma from CAP-exposed mice activates NF-κBα and induces insulin and VEGF resistance, indicating transmission by plasmatic factor(s). To identify putative factors, we exposed lung-specific ecSOD-transgenic (ecSOD-Tg) mice and wild-type (WT) littermates to CAP at concentrations of either ∼60 µg/m^3^ (CAP60) or ∼100 µg/m^3^ (CAP100) and measured the abundance of plasma metabolites by mass spectrometry. In WT mice, both CAP concentrations increased levels of fatty acids such as palmitate, myristate, and palmitoleate and decreased numerous phospholipid species; however, these CAP-induced changes in the plasma lipidome were prevented in ecSOD-Tg mice. Consistent with the literature, we found that fatty acids such as palmitate are sufficient to promote endothelial inflammation. Collectively, our findings suggest that PM_2.5_ exposure, by inducing pulmonary oxidative stress, promotes unique lipidomic changes characterized by high levels of circulating fatty acids, which are sufficient to trigger vascular pathology.

**NEW & NOTEWORTHY** We found that circulating plasma constituents are responsible for air pollution-induced vascular pathologies. Inhalation of fine particulate matter (≤PM_2.5_) promotes a unique form of dyslipidemia that manifests in a manner dependent upon pulmonary oxidative stress. The air pollution-engendered dyslipidemic phenotype is characterized by elevated free fatty acid species and diminished phospholipid species, which could contribute to vascular inflammation and loss of insulin sensitivity.

## INTRODUCTION

Exposure to ambient air pollution is a leading cause of death worldwide and has been linked to 7 million premature deaths globally (1). Exposure to fine particulate matter (PM_2.5_) air pollution appears particularly detrimental to cardiovascular health. In the United States, exposure to elevated levels of PM_2.5_ is associated with 200,000 excessive deaths per year, of which 60%–80% are attributable to cardiovascular disease (CVD) (2–4). Because of this, PM_2.5_ exposure is now recognized as a modifiable risk factor that contributes to cardiovascular morbidity and mortality.

Acute PM_2.5_ exposure increases the risk of myocardial infarction, arrhythmias, and stroke (2), whereas chronic exposure contributes to the development of hypertension, atherosclerosis, and diabetes (2, 4–6). In utero and early life exposure is associated with cardiac dysfunction and the induction of transcriptional as well as metabolic changes in cardiomyocytes (7–10). Because PM_2.5_ exposure affects several aspects of CVD, it is likely that it alters processes that are common to several manifestations of CVD. One such unifying mechanism that could contribute to multiple aspects of CVD is vascular dysfunction. Changes in endothelial reactivity affect not only blood pressure regulation but also atherogenesis, heart failure, and arrhythmogenesis (11). Thus, PM_2.5_-induced endothelial dysfunction could potentially explain several cardiac effects of PM_2.5_ exposure. Nevertheless, the specific processes that trigger PM_2.5_-induced cardiovascular injury and mortality remain unclear.

Several studies (12–15), including our own (16–21), demonstrate that the endothelium is a sensitive target for PM_2.5_ exposure. Chronic PM_2.5_ exposure is associated with persistent endothelial dysfunction (13, 14), and even acute exposure of healthy adults to PM_2.5_ promotes conduit artery vasoconstriction (12), increases blood pressure (15, 22), impairs the levels of circulating endothelial progenitor cells, and induces inflammation and endothelial injury (21, 23). Because the endothelium regulates key processes that contribute to CVD-related pathologies such as atherosclerosis, platelet activation, and thrombosis (11), it is possible that PM_2.5_ exposure increases the risk of developing CVD by impairing endothelial health.

Our previous studies demonstrate that exposure to concentrated ambient PM_2.5_ (CAP) induces vascular inflammation, characterized by activation of the NF-κBα/inflammasome pathway and triggers the development of insulin or VEGF resistance in the blood vessel (17–20). Although PM_2.5_-induced vascular inflammation and insulin resistance occurred in the absence of other metabolic defects, we found that exposure to CAP exacerbates diet-induced systemic insulin resistance and impairs vascular repair mechanisms (17, 20). These findings support the idea that induction of vascular inflammation and growth factor resistance by PM_2.5_ contribute, at least in part, to vascular injury, and could promote the development and progression of type 2 diabetes and CVD. Interestingly, lung-specific overexpression of the antioxidant enzyme extracellular superoxide dismutase (ecSOD) that protects against PM_2.5_-induced pulmonary oxidative stress, preserves vascular VEGF and insulin sensitivity, and prevents vascular inflammation (17, 20), suggesting that pulmonary oxidative stress is a key mediator of PM_2.5_-induced vascular pathologies.

It remains unclear how oxidative stress in the lungs transmits pathological signals that impact blood vessels. A possible means of transmission could be plasmatic factors, which could convey PM_2.5_-induced pulmonary oxidative stress to the circulation, where they trigger vascular inflammation and growth factor resistance. In support of this general idea, exposure to diesel exhaust particles was found to activate the endothelium via a plasmatic factor (24). Nevertheless, the identity of the plasmatic factors that contribute to air pollution-induced vascular inflammation and growth factor resistance remain unknown.

Circulating metabolites may be effective transducers of stress in response to air pollution exposure as likewise shown for other pathologies. For example, free fatty acids have been shown to promote vascular inflammation and insulin resistance in the context of metabolic disease (25, 26). In this study, we examined whether CAP-induced vascular inflammation and vascular growth factor resistance could be mediated by a plasmatic factor. Our findings show that plasmatic factors are sufficient to promote vascular inflammation and impair vascular endothelial growth factor (VEGF) and insulin signaling in the blood vessel. To identify potential plasmatic factors responsible for these vascular defects, we assessed the abundance of plasma metabolites after CAP exposure and found that CAP has robust effects on circulating lipids. Exposure to CAP increased circulating fatty acids, which are known to be sufficient to induce vascular inflammation and insulin resistance. Interestingly, we found that CAP exposure increased free fatty acids and decreased circulating phospholipids, sphingomyelins, and cholesterol, and these changes to the plasma lipidome were completely prevented by pulmonary overexpression of ecSOD. Collectively, these findings indicate that PM_2.5_ exposure alters the plasma metabolome in a manner dependent on the redox state of the lungs and that distinct classes of metabolites, such as fatty acids, may directly impact the health of blood vessels and their signaling responses to growth factors such as insulin and VEGF.

## MATERIALS AND METHODS

### Animals and Exposures

For plasma incubation experiments, 12-wk-old male C57BL/6J mice (Jackson Laboratories, *exposures 1* and *2*) were randomly assigned to three experimental groups (*group 1*: aorta donors, no exposure, naïve; *group 2*: plasma donor, HEPA-filtered air exposure, air; *group 3*: plasma donor, CAP exposure, CAP) and acclimated to our housing conditions for 1 wk. For the metabolomics experiments (*exposures 3* and *4*), male, age-matched, in-house-bred ecSOD-Tg mice (17, 20, 27), and wild-type (WT) littermates were assigned randomly to air and CAP exposure groups. The mice were exposed for 9 consecutive days to either HEPA-filtered air (6 h/day) or CAP (6 h/day) using our versatile aerosol enrichment system (VACES), as described before (17–20). The specific exposure concentrations are summarized in Table 1. On *day 9*, aortas were collected from naïve (unexposed) mice for the plasma incubation experiments. Immediately after the final exposure, blood glucose was measured (Accu-Check glucometer, Aviva, Roche) in the donor mice. Mice were euthanized with sodium pentobarbital (150 mg/kg). Blood was collected via cardiac puncture using Na_4_·EDTA as an anticoagulant, and organs and tissues were excised. Separated plasma and collected tissues were either used immediately (for plasma incubation experiments) or snap-frozen and stored at −80°C. All animal experiments were performed in accordance with the APS’s “Guiding Principles in the Care and Use of Animals,” following protocols approved by the University of Louisville Institutional Animal Care and Use Committee.

**Table 1.** Gravimetric assessment of the exposure concentration

*Exposure*	Experiment	Duration, days	HEPA, μg/m^3^	Ambient, μg/m^3^	CAP, μg/m^3^	Enrichment Factor
*1*	Plasma incubation I	9	1.9	8.8	83.7	5.3-fold
*2*	Plasma incubation II	9	1.6	14.1	74.6	5.3-fold
*3*	Metabolome I, CAP60	9	1.9	8.3	61.8	7.4-fold
*4*	Metabolome II, CAP100	9	8.9	19.0	97.0	5.1-fold

Data for the fine particulate matter (PM_2.5_) concentration in the ambient air, the HEPA-filtered air, and the concentrated ambient PM_2.5_ (CAP) chambers during the specific 9-day exposures. Data are based on gravimetric filter measurements defined as mass divided by air flow (L/min) and the enrichment factor indicates the fold increase by which the versatile aerosol enrichment system (VACES) concentrates PM_2.5_ from the ambient air.

### Plasma and Free Fatty Acid Incubations

To assess whether CAP exposure induces vascular inflammation and impairs vascular VEGF and insulin signaling via a circulating factor, we performed ex vivo plasma incubation experiments following a protocol that was adapted from studies by others (24, 28, 29) and our own experience with isolated aorta experiments (17, 19, 20). For this, we isolated aortas from naïve animals, removed periadventitial connective tissue, and then incubated (1 h; standard cell culture conditions: 5% CO_2_, 37°C) the aortas with plasma immediately after its isolation from air or CAP-exposed mice. The aortas were then stimulated with vehicle (HBSS), insulin (100 nM, 10 min; Humulin-RP, Eli-Lilly), or VEGF (20 ng/mL, 10 min; recombinant murine VEGF165, Peprotech). Aortas were washed in ice-cold PBS, snap-frozen, and stored at −80°C until their analysis by Western blot.

To test for the induction of vascular inflammation by free fatty acids (FFA), we incubated human umbilical vein cells [HUVECs, Promocell, cultured as described (30)] with bovine serum albumin (BSA, vehicle, Sigma-Aldrich) or 100 µM palmitic acid (Sigma-Aldrich, complexed with BSA, PA/BSA) for 1 h. HUVEC incubated with TNF-α (10 ng/mL, 15 min) were used as a positive control. After the treatment, the cells were washed in ice-cold PBS and lysed; lysates were stored at −80°C until their analysis by Western blot, as in Ref. (30).

### Western Blot Analyses

Aortic inflammation and vascular insulin and VEGF signaling were analyzed by performing Western blots to examine IκBα degradation, a marker of NF-κBα activation, and insulin or VEGF-induced Akt phosphorylation (17–20, 31). In addition, IκBα degradation was examined to test for the activation of the NF-κBα pathway by FFA in HUVECs. As described before (17–20, 30, 31), tissue and cells were lysed, and proteins were separated by SDS-PAGE, transferred to PVDF membranes (Bio-Rad, Hercules, CA), and probed with antibodies against IκBα and actin or phospho-Akt (Ser473) and Akt. Antibodies against IκBα, phospho-Akt (Ser473), and Akt (1:1,000) were obtained from Cell Signaling Technology (Danvers, MA), and the actin (1:2,000) antibody was purchased from Sigma-Aldrich. The blots were developed with ECL plus (Amersham Biosciences, Piscataway, NJ; or Pierce, Thermo Fisher, Waltham, MA), and band intensities (Typhoon 9400 variable mode imager Amersham Biosciences; or myECL Imager, Thermo Fisher) were quantified using Image Quant TL software (Amersham Biosciences, Piscataway, NJ; or myImageAnalysis Software, Thermo Fisher).

### Plasma Parameter

Circulating levels of insulin (ALPCO, Salem, NH) and vascular growth factor (VEGF; R&D Systems, Minneapolis, MN) were determined by immunoassay. HOMA-IR and HOMA-β scores were calculated from fasting blood glucose and plasma insulin levels according to the homeostatic model assessment (HOMA).

### Real-Time PCR

Pulmonary mRNA was isolated using the RNeasy Mini Kit (Qiagen), and cDNA was prepared with the iScript cDNA Synthesis Kit (Bio-Rad). Quantitative real-time PCR (qRT-PCR) was performed as described (30) using the following primer sets: soluble superoxide dismutase 1 (*Sod1*): forward primer: 5′- GATGAAGAGAGGCATGTTGGA-3′, reverse primer: 5′- TGTACGGCCAATGATGGAATG-3′; mitochondrial superoxide dismutase 2 (*Sod2*): forward primer: 5′- GCGGTCGTGTAAACCTCAT-3′, reverse primer: 5′- CCAGAGCCTCGTGGTACTTC-3′; extracellular superoxide dismutase 3 (*Sod3*): forward primer: 5′- CTGAGGACTTCCCAGTGAC-3′, reverse primer: 5′- GGTGAGGGTGTCAGAGTGT-3′; catalase (*Cat*): forward primer: 5′- AGCGACCAGATGAAGCAGTG-3′, reverse primer: 5′- TCCGCTCTCTGTCAAAGTGTG-3′; heme oxygenase-1 (*Hmox1*): forward primer: 5′- CACGCATATACCCGCTACCT-3′, reverse primer: 5′- CCAGAGTGTTCATTCGAGA-3′; nuclear factor (erythroid-derived 2)-like 2 (*Nrf2*): forward primer: 5′- CTCGCTGGAAAAAGAAGTG-3′, reverse primer: 5′- CCGTCCAGGAGTTCAGAGG-3′; glutathione *S*-transferase-A (*Gsta*): forward primer: 5′- TGATTGCCGTGGCTCCATTTA-3′, reverse primer: 5′- CAACGAGAAAAGCCTCTCCGT-3′; glutathione *S*-transferase-M (*Gstm*): forward primer: 5′- AGCTCACGCTATTCGGCTG-3′, reverse primer: 5′- GCTCCAAGTATTCCACCTTCAGT-3′; glutathione *S*-transferase-P (*Gstp*): forward primer: 5′- ATGCCACCATACACCATTGTC-3′, reverse primer: 5′- GGGAGCTGCCCATACAGAC-3′; tumor necrosis factor-α (*Tnf*): forward primer: 5′- GCATGATCCGCGACGTGGAA-3′, reverse primer: 5′- AGATCCATGCCGTTGGCCAG-3′; macrophage inflammatory protein-1α (*Ccl3*): forward primer: 5′- ACTGACCTGGAACTGAATGCCTGA-3′, reverse primer: 5′- ATGTGGCTACTTGGCAGCAAACAG-3′; monocyte chemotactic protein-1 (*Ccl2*): forward primer: 5′- ATGCAGGTCCCTGTCATG-3′, reverse primer: 5′- GCTTGAGGTGGTTGTGGA-3′; interleukin-1β (Il1): forward primer: 5′- CTCCATGAGCTTTGTACAAGG-3′, reverse primer: 5′- TGCTGATGTACCAGTTGGGG-3′; and glyceraldehyde 3-phosphate dehydrogenase (*Gapdh*): forward primer: 5′- AGGTCATCCCAGAGCTGAACG-3′, reverse primer: 5′- GGAGTTGCTGTTGAAGTCGCA-3′. The primer set for interleukin-6 (*Il6*) was purchased from SA Bioscience (SABioscience, Qiagen, Valencia, CA).

### Metabolomics

Plasma was stored frozen until metabolite extraction by Metabolon, Inc., and subjected to metabolic profiling by ultrahigh-performance liquid chromatography/tandem mass spectrometry. To remove protein, dissociate small molecules bound to protein or trapped in the precipitated protein matrix, and to recover chemically diverse metabolites, proteins were precipitated with methanol under vigorous shaking for 2 min (Glen Mills GenoGrinder 2000) followed by centrifugation. For quality control, recovery standards were added before the first step in the extraction process. The extracts were divided into several fractions. Two fractions were used for reverse-phase (RP)/UPLC-MS/MS comprising positive ion mode electrospray ionization (ESI) and negative ion mode ESI; one fraction was used for HILIC/UPLC-MS/MS with negative ion mode ESI; and one fraction was reserved for potential later analyses. A cocktail of standards known not to interfere with the measurement of endogenous compounds was spiked into every sample, which helps monitor instrument performance and aids in chromatographic alignment.

All methods used a Waters ACQUITY UPLC and a Thermo Scientific Q-Exactive high-resolution/accurate mass spectrometer interfaced with a heated electrospray ionization (HESI-II) source and Orbitrap mass analyzer operated at 35,000 mass resolution. The sample extract was reconstituted in solvents compatible with each MS/MS method. Each reconstitution solvent contained a series of standards at fixed concentrations to ensure injection and chromatographic consistency. One aliquot was analyzed using acidic positive ion conditions, chromatographically optimized for more hydrophilic compounds. In this method, the extract was gradient eluted from a C18 column (Waters UPLC BEH C18-2.1 × 100 mm, 1.7 µm) using water and methanol, containing 0.05% perfluoropentanoic acid (PFPA) and 0.1% formic acid (FA). Another aliquot was also analyzed using acidic positive ion conditions; however, it was chromatographically optimized for more hydrophobic compounds. In this method, the extract was gradient eluted from the same aforementioned C18 column using methanol, acetonitrile, water, 0.05% PFPA, and 0.01% FA and was operated at an overall higher organic content. Another aliquot was analyzed using basic negative ion optimized conditions using a separate dedicated C18 column. The basic extracts were gradient-eluted using methanol and water, with 6.5 mM ammonium bicarbonate at pH 8. The fourth aliquot was analyzed via negative ionization following elution from a HILIC column (Waters UPLC BEH Amide 2.1 × 150 mm, 1.7 µm) using a gradient consisting of water and acetonitrile, with 10 mM ammonium formate, pH 10.8. The MS analysis alternated between MS and data-dependent MS*^n^* scans using dynamic exclusion. The scan range varied slightly between methods but covered 70–1,000 m/z.

Compounds were identified by comparison to library entries of purified, authenticated standards or recurrent unknown entities, with known retention indices (RI), mass to charge ratios (*m/z*), and chromatographic signatures (including MS/MS spectral data) (Metabolon, Inc.). Biochemical identifications were based on three criteria: retention index within a narrow RI window of the proposed identification, accurate mass match to the library ±10 ppm, and the MS/MS forward and reverse scores between experimental data and authentic standards. Proprietary visualization and interpretation software (Metabolon, Inc.) was used to confirm the consistency of peak identification among the various samples. Library matches for each compound were checked for each sample and corrected, if necessary. Area under the curve was used for peak quantification.

Metabolomic data from two independent exposure studies were analyzed using Metaboanalyst 4.0 software (http://www.metaboanalyst.ca/). Data were analyzed following imputation of values and interquartile range filtering, which identified and removed variables unlikely to be of use when modeling the data. The data were then log-transformed and auto-scaled (mean-centered and divided by the standard deviation of each variable), followed by univariate (e.g., ANOVA contrasts, volcano plots), multivariate (e.g., PLS-DA, importance measures), and cluster (heatmap and dendrogram) analyses.

### Statistical Analyses

Data are reported as means ± SE, unless indicated otherwise. Unpaired Student’s *t* test or ANOVA are used for two group or multiple group comparisons, where appropriate, and *P* < 0.05 was considered significant. For Volcano plot analyses, a raw *P* value <0.05 and a fold change of 1.25 were used as cut-offs. To gain a broad picture of the analytes affected by CAP, metabolites with an adjusted *P* value (false discovery rate, FDR) of *P* < 0.10 were considered statistically significant, and Fisher’s least significant difference test was used for multiple comparisons.

## RESULTS

### Exposure to CAP Induces Vascular Inflammation and Impairs Vascular Growth Factor Signaling via a Plasmatic Factor

**Figure 1.**
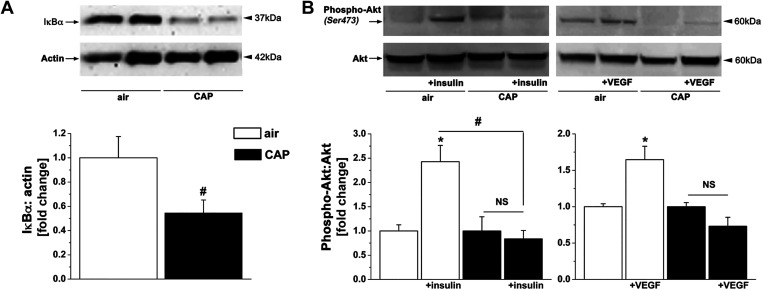
CAP exposure induces vascular injury via a plasmatic factor. Western blot analysis of inflammation (*A*) and insulin/VEGF resistance (*B*) in naïve aortas incubated with plasma isolated from mice exposed for 9 days to HEPA-filtered air or CAP. The aortas were treated with vehicle or stimulated with either insulin (100 nM) or VEGF (20 ng/mL) for 10 min. Western blot data are normalized to the vehicle or air controls. Data are means ± SE (*P < 0.05, insulin/VEGF vs. vehicle; #*P* < 0.05, air vs. CAP, *n* = 4). CAP, concentrated ambient PM_2.5_; NS, not significant; VEGF, vascular endothelial growth factor.

Our previous studies suggest that PM_2.5_ stimulates vascular inflammation and impairs vascular insulin and VEGF signaling by inducing pulmonary oxidative stress (17, 20). However, it is still unclear how the induction of pulmonary oxidative stress transmits systemically to induce vascular injury. To determine whether a circulating factor might be responsible for the vascular effects of PM_2.5_ inhalation, we investigated whether ex vivo treatment of naïve aortas with plasma isolated from CAP-exposed mice induces inflammation (*exposure 1*) and insulin/VEGF resistance (*exposure 2*). Plasma was isolated from mice exposed to a cumulative CAP dose of 83.7 or 74.6 μg/m^3^ in *exposure 1* or *2*, respectively (Table 1). Of note, CAP exposure did not affect circulating levels of insulin or VEGF (Table 2). Naïve aortas were incubated with freshly isolated plasma from air- or CAP-exposed mice for 1 h and then stimulated with either insulin or VEGF for 10 min. Western blot analysis showed that CAP exposure decreased the abundance of IκBα (Fig. 1*A*) and reduced insulin- and VEGF-induced Akt phosphorylation (Fig. 1*B*), indicating activation of the NκBα pathway as well as the induction of insulin and VEGF resistance. These findings support the idea that a plasmatic factor is responsible for the induction of vascular inflammation and insulin and VEGF resistance that manifests with CAP exposure.

**Table 2.** Physiological and plasma parameter in mice used for the plasma incubation experiment (exposure 2)

		Insulin			VEGF		
	Naïve	Air	CAP	*P*	Air	CAP	*P*
BW, g	28.1 ± 0.4	27.3 ± 1.2	26.9 ± 0.3	0.719	28.3 ± 0.7	28.5 ± 1.0	0.815
Heart:BW, %		0.48 ± 0.01	0.48 ± 0.01	0.920	0.50 ± 0.01	0.52 ± 0.02	0.139
Lung:BW, %		0.51 ± 0.01	0.49 ± 0.02	0.287	0.52 ± 0.03	0.52 ± 0.01	0.919
Glucose, mg/dL	182 ± 4	184 ± 15	170 ± 9	0.435	189 ± 11	165 ± 10	0.142
Insulin, ng/mL		0.41 ± 0.01	0.41 ± 0.01	0.637	0.42 ± 0.01	0.40 ± 0.01	0.109
HOMA-IR		5.4 ± 0.5	4.9 ± 0.2	0.393	5.6 ± 0.3	4.8 ± 0.3	0.100
HOMA-β, %		36.6 ± 3.9	40.2 ± 3.3	0.516	35.3 ± 3.3	41.4 ± 3.1	0.225
VEGF, pg/mL		15.3 ± 2.8	15.4 ± 3.7	0.978	19.8 ± 2.0	13.14 ± 2.1	0.065

Values are means ± SE, *n* = 4. Physiological parameters measured in naïve mice (aorta donor) and in mice exposed for 9 days (*exposure 2*) to air or CAP (plasma donor) for the plasma incubation experiment (see Fig. 1*B*). BW, body weight; HOMA-IR, fasting blood glucose (mmol/L) × fasting plasma insulin levels (mU/L)/22.5; HOMA-β, 20× fasting plasma insulin levels (mU/L)/fasting blood glucose (mmol/L)–3.5, %; VEGF, vascular growth factor.

It has been proposed that PM_2.5_-induced oxidative stress is linked mechanistically to cardiac and vascular injury (2, 32–37). Moreover, our previous findings suggest that CAP exposure promotes vascular inflammation and impairs vascular signaling dependent on the induction of pulmonary oxidative stress (17, 20). To confirm CAP-induced pulmonary oxidative stress and adaptive responses, we measured the expression of several antioxidant response genes in the lungs from air and CAP-exposed mice. We found that CAP exposure increased, similar to our previous findings, the mRNA levels of *Sod2* and *Sod3* in lungs (Table 3). We observed no upregulation of inflammatory genes in lungs from CAP-exposed mice (Table 3).

**Table 3.** Expression of antioxidant defense and inflammatory genes in lungs of plasma donor mice that have been exposed for 9 days to air or CAP (exposure 2)

Relative *mRNA*: *Gapdh* (Fold Change)	Air	CAP	*P*
*Sod1*	1.00 ± 0.32	0.78 ± 0.28	0.571
*Sod2*	1.00 ± 0.19	4.24 ± 1.36	0.015
*Sod3*	1.00 ± 0.34	11.10 ± 7.11	0.017
*Cat*	1.00 ± 0.43	0.80 ± 0.32	0.512
*Hmox1*	1.00 ± 0.25	0.87 ± 0.24	0.678
*Nrf2*	1.00 ± 0.36	0.88 ± 0.44	0.571
*Gsta*	1.00 ± 0.53	0.54 ± 0.27	0.212
*Gstm*	1.00 ± 0.25	0.94 ± 0.26	0.910
*Gstp*	1.00 ± 0.56	0.78 ± 0.23	0.623
*Tnfa*	1.00 ± 0.22	1.52 ± 0.73	0.970
*Il1b*	1.00 ± 0.45	0.84 ± 0.19	0.678
*Il6*	1.00 ± 0.23	1.10 ± 0.23	0.791
*Ccl3*	1.00 ± 0.16	0.92 ± 0.14	0.721
*Ccl2*	1.00 ± 0.27	0.63 ± 0.27	0.212

Values are means ± SE normalized to air controls; *n* = 8. Levels of pulmonary *mRNA* were measured in lungs of mice exposed for 9 days to air or CAP (*exposure 2*, plasma donor). *Sod1*, soluble superoxide dismutase 1; *Sod2*, mitochondrial superoxide dismutase 2; *Sod3*, extracellular superoxide dismutase 3, aka *ecSOD*; *Cat*, catalase; *Hmox1*, heme oxygenase-1; *Nrf2*, nuclear factor (erythroid-derived 2)-like 2; *Gsta*, glutathione *S*-transferase-A; *Gstm*, glutathione *S*-transferase-M; *Gstp*, glutathione *S*-transferase-P; *Tnfa*, tumor necrosis factor-α; *Il1b*, interleukin-1b; *Il6*, interleukin-6; *Ccl3*, macrophage inflammatory protein-1α, aka *mip-1α*; *Ccl2*, monocyte chemotactic protein-1, aka *mcp-1*.

### Pulmonary Overexpression of ecSOD Prevents CAP-Induced Changes in the Circulating Metabolome

Our results from plasma incubation experiments, combined with findings from our previous studies that demonstrate that PM_2.5_ by inducing pulmonary oxidative stress stimulates vascular inflammation and insulin/VEGF resistance (17, 20), suggest that PM_2.5_-induced vascular inflammation and insulin/VEGF resistance is caused by a circulating factor and that the generation of this circulating factor is dependent on pulmonary oxidative stress. Hence, we tested how pulmonary oxidative stress contributes to CAP-induced changes in the plasma metabolome. For this, we exposed ecSOD-Tg mice and their WT littermates to HEPA-filtered air or CAP in two independent exposure experiments. Mice were exposed to a cumulative CAP dose (Table 1) of either 61.8 μg/m^3^ (*exposure 3*, Metabolome I, CAP60) or 97.0 μg/m^3^ (*exposure 4*, Metabolome II, CAP100). Immediately after the final exposure, we collected plasma and assessed the relative concentrations of circulating metabolites via untargeted metabolomic analysis. Our previous studies confirmed the overexpression of ecSOD in the lungs and established that this protects from CAP-induced pulmonary oxidative stress (17, 20).

**Figure 2.**
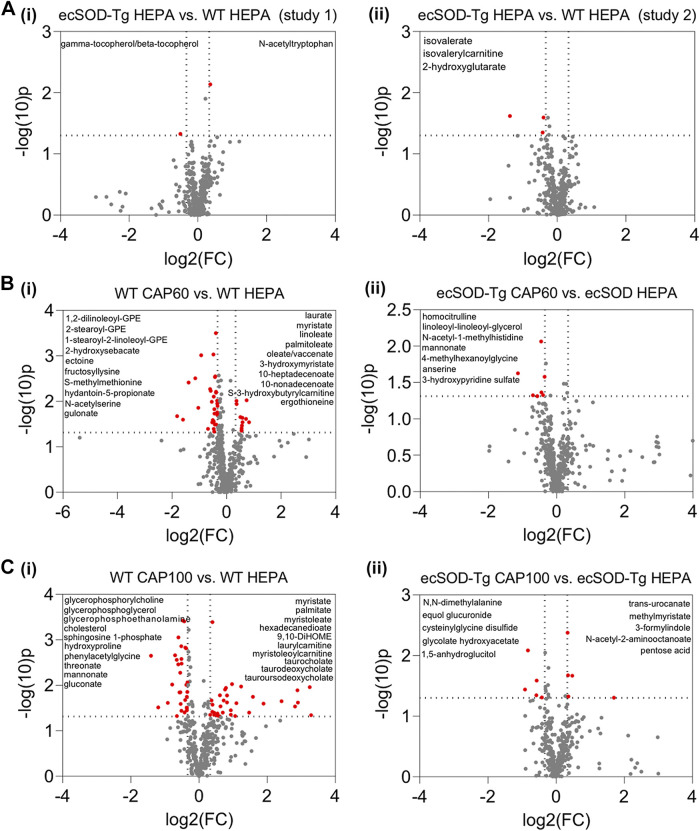
Overexpression of ecSOD in the lung prevents CAP-induced changes in the plasma metabolome. Volcano plots comparing plasma metabolite changes in ecSOD-Tg and WT mice under control conditions (HEPA-filtered air) and after CAP exposure: Mice were subjected to 9 days of exposure (6 h/day) to HEPA-filtered air, ∼60 µg/m^3^ CAP (CAP60, *exposure 3*), or ∼100 µg/m^3^ CAP (CAP100, *exposure 4*). Relative metabolite abundances in plasma were measured by unbiased metabolomic profiling. Metabolites that changed at least 1.25-fold with a raw *P* value <0.05 were considered statistically significant (indicated in red). The identities of some of the metabolites that changed significantly with CAP exposure are provided in the upper left and/or right quadrants of each volcano plot. *A*: plasma metabolite changes caused by ecSOD overexpression alone in metabolomics *study I* (*i*; *n* = 15 mice, 9 WT mice and 6 ecSOD-Tg mice) and metabolomics *study II* (*ii*; *n* = 19 mice, 10 WT mice and 9 ecSOD-Tg mice). *B*: plasma metabolite changes caused by CAP60 exposure in WT mice (*i*; *n* = 17 mice, 8 WT CAP60, 9 WT HEPA) and ecSOD-Tg mice (*ii*; *n* = 15 mice, 9 ecSOD-Tg CAP60 and 6 ecSOD-Tg HEPA). *C*: plasma metabolite changes caused by CAP100 exposure in WT mice (*i*; *n* = 19 mice, 9 WT CAP100 and 10 WT HEPA) and ecSOD-Tg mice (*ii*; *n* = 19 mice, 10 ecSOD-Tg CAP100 and 9 ecSOD-Tg HEPA). CAP, concentrated ambient PM_2.5_; ecSOD-Tg, extracellular superoxide dismutase-transgenic; WT, wild type.

To gain insight into metabolic changes caused by PM_2.5_ exposure in WT mice, we assessed the relative concentrations of circulating metabolites via untargeted metabolomic analyses. We then compared the metabolomic changes that occurred in the plasma collected from WT and ecSOD-Tg mice exposed to either HEPA-filtered air, CAP60, or CAP100. Overexpression of ecSOD in the lungs had minimal effects on the plasma metabolome in control mice inhaling HEPA-filtered air, with only five metabolites differing from WT controls (Fig. 2*A*). In WT mice, we found 45 plasma metabolites that were elevated by CAP60 exposure by at least 1.25-fold (raw *P* value ≤ 0.05). Of the metabolites elevated in WT mice exposed to CAP60, 16 were in the lipid superfamily, with numerous saturated (e.g., laurate, myristate, 3-hydroxymyristate) and unsaturated (e.g., palmitoleate, linoleate, oleate/vaccinate) fatty acids increasing with CAP exposure. Significantly decreased by CAP60 exposure were several GPE species as well as other metabolites (e.g., 2-hydroxysebacate, ectoine, fructosyllysine, and gulonate) (Fig. 2*Bi*). In ecSOD-Tg mice exposed to CAP60, only seven metabolites were significantly different in plasma from CAP60-exposed mice (Fig. 2*Bii*).

Exposure to a higher CAP concentration (CAP100) significantly altered the abundance of 63 metabolites in WT mice (Fig. 2*Ci*), with 29 significantly different metabolites derived from the lipid family. In particular, CAP100 increased saturated fatty acid species (e.g., myristate, palmitate, 3-hydroxylaurate, 3-hydroxystearate, and 3-hydroxymyristate) as well as augmented levels of several unsaturated fatty acids (e.g., palmitoleate, myristoleate, stearidonate, and tetradecadienoate); increased concentrations of dicarboxylate fatty acid species (e.g., hexadecanedioate, octadecenedioate, and 9,10-diHOME); augmented laurylcarnitine, myristoleoylcarnitine, and palmitoleoylcarnitine levels; and elevated the abundance of circulating bile acid metabolites (e.g., taurocholate, taurodeoxycholate, taurochenodeoxycholate, tauroursodeoxycholate, 12-ketolithocholate, and among others). Exposure to CAP100 diminished cholesterol and several phospholipid species including glycerophosphorylcholine, glycerophosphoglycerol, and glycerophosphorylethanolamine. Other metabolites diminished by CAP100 include sphingosine 1-phosphate, hydroxyproline, and several sugar acid species (e.g., threonate, mannonate, and gluconate). Similar to the CAP60 study results, the metabolic features of the CAP-induced dyslipidemia found in WT mice exposed to CAP100 were largely absent in ecSOD-Tg mice: in ecSOD-Tg mice, only 10 metabolites were significantly altered by CAP100 (Fig. 2*Cii*). Collectively, these findings indicate that oxidative stress in the lungs reproducibly drives the acquisition of CAP-induced dyslipidemia, which is characterized by high levels of circulating fatty acids, acylcarnitines and bile acids, and low levels of circulating phospholipids.

### High Concentrations of CAP Drive More Severe Changes in the Circulating Lipidome

**Figure 3.**
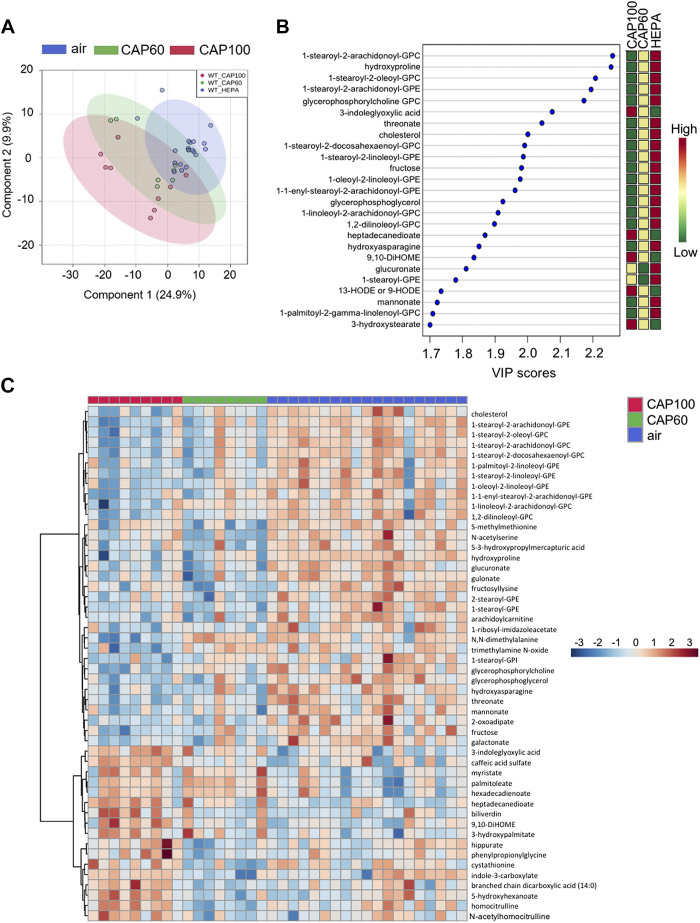
Merged plasma metabolomic data reveal lipid species that are strongly influenced by CAP. Multivariate and heatmap metabolomic analyses of plasma from WT mice exposed to HEPA-filtered air, 60 µg/m^3^ CAP (CAP60) or 100 µg/m^3^ CAP (CAP100) for 9 days (6 h/day). Data from metabolomics *study 1* (*exposure 3*) and metabolomics *study 2* (*exposure 4*) were merged and each biochemical was rescaled to set the median equal to 1. Then, missing values were imputed with the minimum value for each biochemical. *A*: partial least squared discriminant analysis. *B*: variable importance in projection (VIP) analysis. *C*: heatmap analysis showing the 50 most significantly changed plasma metabolites in CAP-exposed mice (ANOVA). An FDR of <0.10 was considered statistically significant (*n* = 36 WT mice: 19 HEPA, 8 CAP60, and 9 CAP100). CAP, concentrated ambient PM_2.5_; FDR, false discovery rate; WT, wild type.

**Figure 4.**
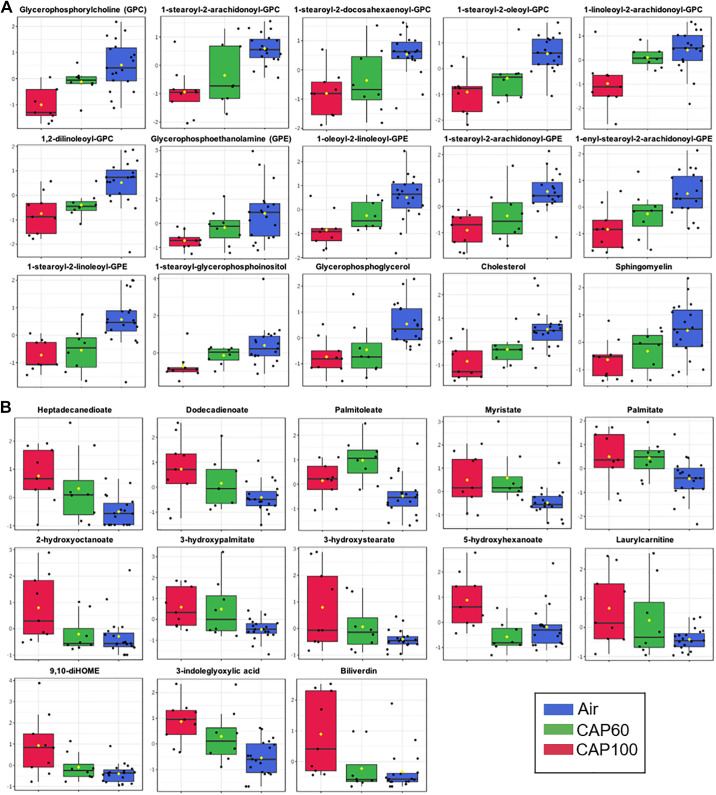
CAP concentration influences the circulating metabolome. Box plots of circulating metabolites that were significantly different in WT mice exposed to HEPA-filtered air, 60 µg/m^3^ CAP (CAP60) or 100 µg/m^3^ CAP (CAP100) for 9 days (6 h/day). Data from metabolomics *study 1* (*exposure 3*) and metabolomics *study 2* (*exposure 4*) were merged and each biochemical was rescaled to set the median equal to 1. Then, missing values were imputed with the minimum value for each biochemical. *A*: lipid species that decreased progressively with increasing CAP exposure. *B*: lipid species that increased progressively with increasing CAP exposure. ANOVA: an FDR of <0.10 was considered statistically significant (*n* = 36 WT mice: 19 HEPA, 8 CAP60, and 9 CAP100). CAP, concentrated ambient PM_2.5_; FDR, false discovery rate; WT, wild type.

Metabolomics analysis of merged data from the CAP60 and CAP100 exposure studies revealed that CAP exposure significantly altered the levels of 96 out of the 620 metabolites (Table 4). Partial least squared discriminant analysis (PLS-DA) implies separation of the groups based on the level of CAP exposure (Fig. 3*A*), and variable importance in projection (VIP) scoring indicated that many of the metabolites driving group separation were lipid species (Fig. 3*B*). Heatmap analysis also demonstrated that lipids are the most prominent metabolite species altered by CAP exposure. Cholesterol and numerous phospholipids were lower in both the CAP60 and CAP100 groups, whereas fatty acids and hydroxylated fatty acids (e.g., myristate, oleate, palmitoleate, and 3-hydroxypalmitate) were higher. In addition, bioactive mono- and dihydroxy fatty acids derived from linoleic acid such as 9- or 13-HODE, 9,10-diHOME, and 12,13-diHOME were elevated in plasma from CAP-exposed mice (Figs. 3*C* and 4 and Table 4).

**Table 4.** Significant CAP-exposure induced changes in plasma metabolites in WT mice

Metabolite	*F* Value	*P* Value	−10 log(*p*)	FDR	Fisher’s LSD
Hydroxyproline	15.722	1.60E-05	4.7959	0.007439	air vs. CAP100; air vs. CAP60
1-Stearoyl-2-arachidonoyl-GPC 18:0/20:4	13.347	5.66E-05	4.2473	0.009064	air vs. CAP100; air vs. CAP60
1-Stearoyl-2-oleoyl-GPC 18:0/18:1	12.374	9.78E-05	4.0099	0.009064	air vs. CAP100; air vs. CAP60
1-stearoyl-2-arachidonoyl-GPE 18:0/20:4	12.062	0.000117	3.9319	0.009064	air vs. CAP100; air vs. CAP60
Homocitrulline	11.736	0.000141	3.8497	0.009064	*CAP100 vs. CAP60* air vs. CAP100; air vs. CAP60
*N*,*N*-dimethylalanine	11.628	0.000151	3.8224	0.009064	air vs. CAP100; air vs. CAP60
Glycerophosphorylcholine (GPC)	11.478	0.000164	3.7841	0.009064	*CAP100 vs. CAP60* air vs. CAP100; air vs. CAP60
2-Stearoyl-GPE 18:0	11.342	0.000178	3.7492	0.009064	air vs. CAP100; air vs. CAP60
Glucuronate	11.196	0.000194	3.7114	0.009064	air vs. CAP100; air vs. CAP60
Fructosyllysine	11.182	0.000196	3.7078	0.009064	*CAP100 vs. CAP60* air vs. CAP100; air vs. CAP60
*N*-acetylserine	11.032	0.000214	3.6687	0.009064	*CAP100 vs. CAP60* air vs. CAP100; air vs. CAP60
1-Stearoyl-GPE 18:0	10.727	0.000258	3.5889	0.009986	air vs. CAP100; air vs. CAP60
Threonate	10.165	0.000363	3.4396	0.012482	air vs. CAP100; air vs. CAP60
1-Stearoyl-2-linoleoyl-GPE 18:0/18:2	10.065	0.000387	3.4126	0.012482	air vs. CAP100; air vs. CAP60
*S*-methylmethionine	9.9783	0.000408	3.3892	0.012482	*CAP100 vs. CAP60* air vs. CAP100; air vs. CAP60
3-Indoleglyoxylic acid	9.8966	0.000429	3.3671	0.012482	air vs. CAP100; air vs. CAP60
Trimethylamine N-oxide	9.0477	0.000737	3.1328	0.019097	*CAP100 vs. CAP60* air vs. CAP100
Cholesterol	8.9272	0.000796	3.0989	0.019097	air vs. CAP100; air vs. CAP60
1-Stearoyl-2-docosahexa enoyl-GPC 18:0/22:6	8.9252	0.000797	3.0984	0.019097	air vs. CAP100; air vs. CAP60
Palmitoleate 16:1n7	8.8794	0.000821	3.0855	0.019097	*CAP100 vs. CAP60* air vs. CAP100; air vs. CAP60
Glycerophosphoglycerol	8.591	0.000992	3.0036	0.021015	air vs. CAP100; air vs. CAP60
1-Linoleoyl-2-arachidonoyl-GPC 18:2/20:4n6	8.5232	0.001037	2.9842	0.021015	*CAP100 vs. CAP60* air vs. CAP100
Fructose	8.4745	0.001071	2.9702	0.021015	air vs. CAP100; air vs. CAP60
1-Oleoyl-2-linoleoyl-GPE 18:1/18:2	8.4553	0.001085	2.9647	0.021015	air vs. CAP100; air vs. CAP60
Gulonate	8.3262	0.001182	2.9275	0.021978	air vs. CAP100; air vs. CAP60
1-1-Enyl-stearoyl-2-arachidonoyl-GPE P-18:0/20:4	8.2655	0.00123	2.91	0.022003	air vs. CAP100; air vs. CAP60
1,2-Dilinoleoyl-GPC 18:2/18:2	7.9573	0.001513	2.8203	0.025873	air vs. CAP100; air vs. CAP60
Cystathionine	7.9136	0.001558	2.8074	0.025873	*CAP100 vs. CAP60* air vs. CAP60
9,10-DiHOME	7.4997	0.002066	2.6849	0.033124	*CAP100 vs. CAP60* air vs. CAP100
Heptadecanedioate C17-DC	7.3354	0.002314	2.6357	0.034457	air vs. CAP100; air vs. CAP60
Indole-3-carboxylate	7.312	0.002352	2.6287	0.034457	*CAP100 vs. CAP60* air vs. CAP60
Hydroxyasparagine	7.3	0.002371	2.625	0.034457	*CAP100 vs. CAP60* air vs. CAP60
*N*-acetylcitrulline	7.1256	0.002677	2.5723	0.037723	*CAP100 vs. CAP60* air vs. CAP100
5-Hydroxyhexanoate	6.8747	0.003193	2.4958	0.043283	*CAP100 vs. CAP60* air vs. CAP100
*N*-acetylhomocitrulline	6.8462	0.003258	2.4871	0.043283	*CAP100 vs. CAP60* air vs. CAP100
Arachidoylcarnitine C20	6.7083	0.003592	2.4446	0.046399	air vs. CAP100; air vs. CAP60
1-Palmitoyl-2-linoleoyl-GPE 16:0/18:2	6.5395	0.004052	2.3923	0.050927	air vs. CAP100; air vs. CAP60
1-Ribosyl-imidazoleacetate	6.4757	0.004242	2.3724	0.051906	air vs. CAP100; air vs. CAP60
Mannonate	6.2803	0.004884	2.3113	0.058228	air vs. CAP100; air vs. CAP60
Biliverdin	6.2295	0.005067	2.2953	0.058902	*CAP100 vs. CAP60* air vs. CAP100
Galactonate	6.1126	0.005517	2.2583	0.061149	air vs. CAP100; air vs. CAP60
3-Hydroxypalmitate	6.1111	0.005523	2.2578	0.061149	air vs. CAP100; air vs. CAP60
Oleate/vaccenate 18:1	6.0668	0.005705	2.2438	0.06169	*CAP100 vs. CAP60* air vs. CAP60
Myristate 140	5.9848	0.006058	2.2177	0.064022	air vs. CAP100; air vs. CAP60
1-Stearoyl-2-oleoyl-GPG 18:0/18:1	5.9103	0.006399	2.1939	0.066123	*CAP100 vs. CAP60* air vs. CAP60
13-HODE or 9-HODE	5.8315	0.006782	2.1687	0.068554	air vs. CAP100; air vs. CAP60
12,13-DiHOME	5.7508	0.007199	2.1427	0.0695	*CAP100 vs. CAP60* air vs. CAP100
1-Stearoyl-2-docosahexa enoyl-GPE 18:0/22:6	5.7489	0.007209	2.1421	0.0695	air vs. CAP100; air vs. CAP60
10-Heptadecenoate 17:1n7	5.7277	0.007324	2.1353	0.0695	*CAP100 vs. CAP60* air vs. CAP60
1-Palmitoyl-2-γ-linolenoyl-GPC 16:0/18:3n6	5.6091	0.007999	2.0969	0.074017	air vs. CAP100
3-Hydroxystearate	5.5838	0.008152	2.0887	0.074017	*CAP100 vs. CAP60* air vs. CAP100
2-Oxoadipate	5.5634	0.008277	2.0821	0.074017	air vs. CAP100; air vs. CAP60
10-Nonadecenoate 19:1n9	5.5289	0.008494	2.0709	0.074519	air vs. CAP60
Taurocholate	5.4693	0.008882	2.0515	0.076485	*CAP100 vs. CAP60* air vs. CAP100
Taurodeoxycholate	5.4181	0.00923	2.0348	0.078039	*CAP100 vs. CAP60* air vs. CAP100
*S*-3-hydroxypropyl mercapturicacid (HPMA)	5.3892	0.009434	2.0253	0.078333	air vs. CAP100; air vs. CAP60
3-Phenylpropionate (hydrocinnamate)	5.2757	0.010279	1.988	0.083856	*CAP100 vs. CAP60* air vs. CAP100
3-Hydroxymyristate	5.2001	0.010886	1.9631	0.086605	air vs. CAP100; air vs. CAP60
*S*-3-hydroxybutyrylcarnitine	5.1533	0.011281	1.9477	0.086605	air vs. CAP100; air vs. CAP60
2-Methylbutyrylcarnitine C5	5.1156	0.01161	1.9352	0.086605	*CAP100 vs. CAP60* air vs. CAP100
1-Linoleoyl-2-linolenoyl-GPC 18:2/18:3	5.1087	0.011671	1.9329	0.086605	air vs. CAP100; air vs. CAP60
Malonate	5.0925	0.011816	1.9275	0.086605	*CAP100 vs. CAP60* air vs. CAP60
3-Hydroxylaurate	5.0628	0.012088	1.9176	0.086605	air vs. CAP100; air vs. CAP60
Lyxonate	5.0218	0.012474	1.904	0.086605	air vs. CAP100; air vs. CAP60
5-Hydroxylysine	5.0204	0.012487	1.9035	0.086605	air vs. CAP100; air vs. CAP60
Linoleate 18:2n6	5.013	0.012558	1.9011	0.086605	*CAP100 vs. CAP60* air vs. CAP60
10-Undecenoate 11:1n1	4.9814	0.012866	1.8905	0.086605	air vs. CAP100; air vs. CAP60
Dodecadienoate 12:2	4.973	0.012949	1.8877	0.086605	air vs. CAP100
Hexadecadienoate16:2n6	4.9416	0.013266	1.8772	0.086605	air vs. CAP100; air vs. CAP60
*N*^2^,*N*^2^-dimethylguanosine	4.8918	0.013785	1.8606	0.086605	*CAP100 vs. CAP60* air vs. CAP100
Sphingomyelin d18:2/18:1	4.8889	0.013816	1.8596	0.086605	air vs. CAP100; air vs. CAP60
Linolenate 18:3n3 or 3n6	4.8772	0.013942	1.8557	0.086605	air vs. CAP100; air vs. CAP60
Glycerophosphoethanolamine	4.8671	0.014051	1.8523	0.086605	air vs. CAP100
1-Stearoyl-GPI 18:0	4.86	0.014127	1.8499	0.086605	air vs. CAP100
16-Hydroxypalmitate	4.8524	0.014211	1.8474	0.086605	air vs. CAP100
*N*-formylmethionine	4.8456	0.014285	1.8451	0.086605	air vs. CAP100; air vs. CAP60
Carotene diol 1	4.8339	0.014415	1.8412	0.086605	*CAP100 vs. CAP60* air vs. CAP60
Myristoleate 14:1n5	4.8131	0.01465	1.8342	0.086605	air vs. CAP100; air vs. CAP60
3-Hydroxydecanoate	4.8074	0.014714	1.8323	0.086605	air vs. CAP100; air vs. CAP60
Hippurate	4.754	0.015336	1.8143	0.089138	*CAP100 vs. CAP60* air vs. CAP100
Eicosenoate 20:1n9 or 1n11	4.7034	0.015951	1.7972	0.091568	air vs. CAP60
Cinnamate	4.6483	0.016651	1.7786	0.092252	*CAP100 vs. CAP60* air vs. CAP100
Stearidonate 18:4n3	4.6429	0.016722	1.7767	0.092252	air vs. CAP100; air vs. CAP60
2-Hydroxyoctanoate	4.6185	0.017043	1.7685	0.092252	*CAP100 vs. CAP60* air vs. CAP100
Phenylpyruvate	4.6169	0.017064	1.7679	0.092252	*CAP100 vs. CAP60* air vs. CAP100
Laurylcarnitine C12	4.5871	0.017467	1.7578	0.092252	air vs. CAP100; air vs. CAP60
Hexadecanedioate C16	4.5828	0.017525	1.7563	0.092252	air vs. CAP100; air vs. CAP60
Palmitate 160	4.5802	0.017561	1.7554	0.092252	air vs. CAP100; air vs. CAP60
*N*-acetyl-β-alanine	4.5712	0.017686	1.7524	0.092252	*CAP100 vs. CAP60* air vs. CAP60
Gluconate	4.5466	0.018029	1.744	0.092252	air vs. CAP100; air vs. CAP60
2- or 3-Decenoate 10:1n7 or n8	4.5449	0.018054	1.7434	0.092252	air vs. CAP100
Palmitoleoylcarnitine C16:1	4.5184	0.018433	1.7344	0.093169	air vs. CAP100; air vs. CAP60
Myristoleoylcarnitine C14:1	4.4885	0.01887	1.7242	0.094351	air vs. CAP100; air vs. CAP60
Formiminoglutamate	4.4746	0.019079	1.7194	0.09438	*CAP100 vs. CAP60* air vs. CAP60
3-Methoxytyrosine	4.438	0.019636	1.7069	0.096114	*CAP100 vs. CAP60* air vs. CAP60
*N*-acetylglutamate	4.4233	0.019865	1.7019	0.09622	air vs. CAP60

Metabolites extracted from plasma of male wild-type mice inhaling CAP or HEPA-filtered air for 6 h/day for 9 days. Metabolites were subjected to LC-MS analysis. Metabolites with an adjusted *P* value (FDR) cutoff of *P* < 0.1 were considered significantly different. Fisher’s least significant difference (LSD) method was used for multiple comparisons. CAP, concentrated ambient PM_2.5_; FDR, false discovery rate.

Higher concentrations of CAP augmented metabolite changes in plasma. For instance, exposure to either CAP60 or CAP100 decreased the abundance of GPC, GPE, and glycerophosphoinositol (GPI) species as well as cholesterol and sphingomyelin, an effect that appears stronger with CAP100 than with CAP60 exposure (Fig. 4*A*). Nonhydroxylated and hydroxylated fatty acids, inflammatory lipids, and acylcarnitine species increased with the higher CAP dose (Fig. 4*A*). Collectively, these data suggest that CAP concentration influences the magnitude by which CAP increases levels of circulating fatty acids and decreases levels of phospholipids, cholesterol, and sphingomyelins.

### Treatment with Fatty Acids Induces Endothelial Inflammation

**Figure 5.**
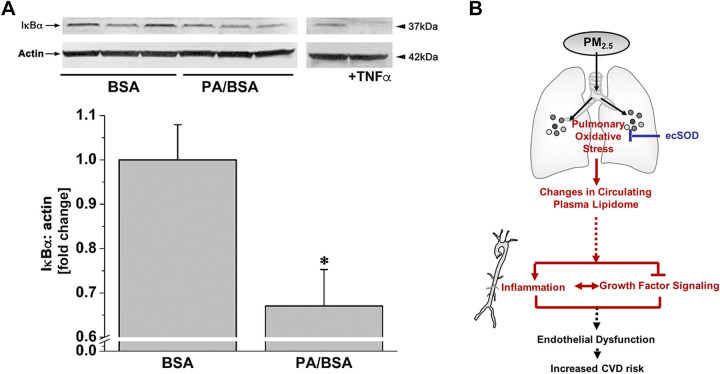
Free fatty acids are sufficient to cause vascular pathology. Western blot analysis of IκBα in endothelial cells (*A*) incubated for 1 h with either bovine serum albumin (BSA, vehicle) or 100 µM palmitic acid (Sigma-Aldrich, complexed with BSA, PA/BSA). HUVEC incubated with TNF-α (10 ng/mL, 15 min) was used as a positive control. Western blot data are normalized to the vehicle controls. Data are means ± SE (*P < 0.05, PA/BSA vs. BSA, *n* = 3). *B*: inhalation of PM_2.5_ air pollution promotes a unique form of dyslipidemia that manifests in a manner dependent on pulmonary oxidative stress. This dyslipidemic phenotype is characterized by diminished phospholipid species and elevated free fatty acid species. Because elevated free fatty acids are sufficient to cause vascular inflammation and insulin or VEGF resistance, it is likely that PM_2.5_-induced dyslipidemia contributes to the increased CVD risk associated with air pollution. BSA, bovine serum albumin; HUVEC, human umbilical vein cell.

Previous studies indicate that circulating free fatty acids promote vascular inflammation and impair insulin signaling (38–42). The influence of fatty acids on vascular inflammation was confirmed by our experiment in endothelial cells, which demonstrated that incubation with palmitic acid (PA) decreases the abundance of IκBα (Fig. 5*A*).

Taken together, exposure to PM_2.5_ induces changes to the plasma metabolome that are dependent on pulmonary oxidative stress. This CAP-induced dyslipidemic phenotype is characterized by an increase in free fatty acids and a decrease in phospholipid. As elevated free fatty acids have been demonstrated as sufficient to cause vascular inflammation and growth factor resistance, it is likely that PM_2.5_-induced dyslipidemia contributes to the increased CVD risk associated with air pollution exposure as summarized in Fig. 5*B*.

## DISCUSSION

Our results indicate that exposure to PM_2.5_ air pollution induces vascular inflammation as well as vascular insulin and VEGF resistance via a plasmatic factor. We find that PM_2.5_ exposure leads to the development of a dyslipidemic phenotype dependent on CAP-induced oxidative stress in the lungs. This pollution-induced form of dyslipidemia is characterized by high levels of circulating FFAs and acylcarnitines and low levels of phospholipid species. We also find that exposure of endothelial cells to heightened levels of fatty acids, such as occurs in vivo with PM_2.5_ exposure, is sufficient to promote inflammation. Taken as a whole, these findings suggest that PM_2.5_-induced elevations in circulating fatty acids may contribute to vascular pathology caused by air pollution.

These findings add to our understanding of the mechanisms by which air pollution promotes vascular injury and dysfunction. Our previous studies demonstrate that exposure to CAP activates NF-κB and inflammasome pathways in blood vessels and impairs vascular insulin and VEGF signaling (17–20). Our findings also suggest that pulmonary oxidative stress contributes to the CAP-induced vascular pathology because lung-specific overexpression of ecSOD prevents PM_2.5_-induced vascular inflammation and growth factor resistance (17, 20). That CAP induces pulmonary expression of *Sod2* and *Sod3*, which encodes the mitochondrial and extracellular isoforms of SOD, in the absence of upregulation of genes encoding other sensitive antioxidant enzyme such as HO-1 and catalase suggests a unique antioxidant response caused by air pollution. This unique response seems to have translational relevance as exposure to elevated levels of PM_2.5_ upregulates ecSOD in human plasma (43).

Our studies also identify a unique form of dyslipidemia caused by PM_2.5_. We find that CAP diminishes levels of numerous phospholipid species, cholesterol, and sphingomyelin, which is similar to the serum metabolomic changes reported after airway administration of a water-soluble PM_2.5_ extract (44). CAP also increased the levels of circulating FFAs such as myristate, palmitate, and palmitoleate, among others, in a manner dependent on CAP concentration. This is interesting given the fact that many fatty acid species have been shown to be sufficient causes of vascular and endothelial inflammation and insulin resistance (38–42, 45, 46). That these metabolic changes in plasma could contribute to vascular inflammation and growth factor resistance are demonstrated by our ex vivo incubation experiments in which plasma from CAP-exposed mice promoted NF-κB activation and insulin and VEGF resistance. Such findings are congruent with those that find that particulate air pollution from diesel exhaust activates the endothelium via a plasmatic factor (24). This study demonstrated that a 24-h treatment of ECs with plasma of individuals exposed to diesel emission induces inflammation. Studies from the same group demonstrated that infusion of serum from ozone-exposed rats for 30 min impaired Ach-mediated vasodilation, indicating that short-term treatment with plasma isolated from air pollution-exposed rodents triggers vascular dysfunction (29, 47). The results of our isolated aorta study similarly indicate that the induction of vascular inflammation and insulin resistance is an immediate response to a PM_2.5_-induced plasmatic factor (e.g., FFA). This is further supported by a study showing that short-term FFA incubation (3 h) induces vascular inflammation and insulin resistance (28). However, processes contributing to PM_2.5_-induced generation and plasmatic accumulation of FFA in vivo might take longer (e.g., days) and require further investigation. Collectively, our findings help develop a working model suggesting that CAP-induced vascular toxicity is initiated via oxidative stress in the lungs, which elevates levels of circulating FFAs and promotes vascular inflammation and growth factor resistance (Fig. 5*B*).

It remains unclear how PM_2.5_-induced oxidative stress in the lungs elevates circulating fatty acids and diminishes phospholipids, cholesterol, and sphingomyelins. Possibilities supported by the literature include activation of lipolysis, dysregulation of lipid metabolism in the liver, and/or mitochondrial dysfunction. Supporting the idea that lipolysis may be involved are studies showing that exposure to air pollution elevates catecholamines (48–50), which are potent activators of lipolysis enzymes (51) and could lead to the development of CAP-induced dyslipidemia. The potential involvement of the liver is supported by numerous studies showing that PM_2.5_ promotes liver steatosis and fibrosis (52–55). In addition, we found that biliverdin was elevated in mice exposed to the higher level of CAP, which may suggest some degree of liver damage. The idea that mitochondrial damage occurs with CAP exposure is supported by in vivo stable isotope metabolomics data, which indicates decreased liver Krebs cycle activity in mice exposed to CAP (56). That circulating acylcarnitines—known markers of defects in fatty acid oxidation (57)—were elevated by CAP in our study further attests to the plausibility of this mechanism of CAP-induced dyslipidemia. Future studies are required to delineate the contribution of each of these possibilities to CAP-induced changes in the circulating lipidome.

Beyond changes in simple saturated and unsaturated fatty acids, phospholipids, and cholesterol, several other metabolites were found to be significantly altered by CAP. For example, toxic hydroxylated fatty acids such as 9,10-diHOME were elevated by CAP, which could influence the onset or progression of PM_2.5_-induced pathology; 9,10-diHOME is particularly interesting because it is formed from a cytochrome P450-derived epoxide via soluble epoxide hydrolase, a metabolic conversion that has been suggested to occur in neutrophils (58, 59). Thus, it is possible that neutrophils, which are increased in the lungs with PM_2.5_ exposure (53), may stimulate pulmonary redox changes that provoke CAP-induced vascular pathologies. In mice exposed to higher levels of CAP, we also found an elevation in numerous bile acid species. These species were not changed at the lower level of CAP exposure, which suggests a threshold effect of CAP on bile acids. Bile acids may be important for regulating systemic lipid metabolism and the development of insulin resistance (60). Decreases in sugar acids were also prominent at the higher level of CAP exposure; it remains unclear why these metabolites decrease with CAP and what their significance to particle toxicity may be, if any. Regardless, the fact that ecSOD overexpression blocked CAP-induced dyslipidemia and other changes in the plasma metabolome suggests that pulmonary oxidative stress is a nidus for the systemic metabolic effects of PM_2.5_.

Our study has several limitations. Although it is known that elevated saturated fatty acids are sufficient causes of endothelial inflammation and growth factor resistance, it is possible that other circulating factors contribute to PM_2.5_-induced vascular toxicity. One possibility is that circulating cytokines could contribute to vascular injury caused by CAP; however, in past studies, we have not found changes in circulating cytokines such as TNF-α and IL-6 in the plasma from CAP-exposed mice (20). In contrast, our studies and investigations by others indicate that PM_2.5_ exposure increases local inflammation in vascular and cardiac tissue (10, 19). Another possibility is that circulating microparticles or exosomes could deliver cargo to the vasculature that contributes to pathology; few published reports have addressed this possibility (23, 61, 62). Nevertheless, our findings suggest that PM_2.5_ promotes a unique form of dyslipidemia that is dependent on pulmonary oxidative stress which could contribute to the etiology and progression of vascular disease.

## GRANTS

This work was supported by grants from the National Institute of Health: ES027881, ES028268, and GM127607.

## DISCLOSURES

No conflicts of interest, financial or otherwise, are declared by the authors.

## AUTHOR CONTRIBUTIONS

B.G.H. and P.H. conceived and designed research; B.R., A.R., and P.H. performed experiments; B.G.H., B.R., A.R., and P.H. analyzed data; B.G.H. and P.H. interpreted results of experiments; B.G.H., A.R., and P.H. prepared figures; B.G.H. and P.H. drafted manuscript; B.G.H. and P.H. edited and revised manuscript; B.G.H., B.R., A.R., and P.H. approved final version of manuscript.
